# The Value of Extracting Clinician-Recorded Affect for Advancing Clinical Research on Depression: Proof-of-Concept Study Applying Natural Language Processing to Electronic Health Records

**DOI:** 10.2196/34436

**Published:** 2022-05-12

**Authors:** Vanessa Panaite, Andrew R Devendorf, Dezon Finch, Lina Bouayad, Stephen L Luther, Susan K Schultz

**Affiliations:** 1 Research Service James A. Haley Veterans' Hospital Tampa, FL United States; 2 Department of Psychology University of South Florida Tampa, FL United States; 3 Department of Information Systems and Business Analytics Florida International University Miami, FL United States; 4 College of Public Health University of South Florida Tampa, FL United States; 5 Mental Health and Behavioral Sciences James A. Haley Veterans' Hospital Tampa, FL United States; 6 Department of Psychiatry and Behavioral Neurosciences University of South Florida Tampa, FL United States

**Keywords:** depression, affect, natural language processing, electronic health records, vocabularies

## Abstract

**Background:**

Affective characteristics are associated with depression severity, course, and prognosis. Patients’ affect captured by clinicians during sessions may provide a rich source of information that more naturally aligns with the depression course and patient-desired depression outcomes.

**Objective:**

In this paper, we propose an information extraction vocabulary used to pilot the feasibility and reliability of identifying clinician-recorded patient affective states in clinical notes from electronic health records.

**Methods:**

Affect and mood were annotated in 147 clinical notes of 109 patients by 2 independent coders across 3 pilots. Intercoder discrepancies were settled by a third coder. This reference annotation set was used to test a proof-of-concept natural language processing (NLP) system using a named entity recognition approach.

**Results:**

Concepts were frequently addressed in templated format and free text in clinical notes. Annotated data demonstrated that affective characteristics were identified in 87.8% (129/147) of the notes, while mood was identified in 97.3% (143/147) of the notes. The intercoder reliability was consistently good across the pilots (interannotator agreement [IAA] >70%). The final NLP system showed good reliability with the final reference annotation set (mood IAA=85.8%; affect IAA=80.9%).

**Conclusions:**

Affect and mood can be reliably identified in clinician reports and are good targets for NLP. We discuss several next steps to expand on this proof of concept and the value of this research for depression clinical research.

## Introduction

### Background

Depression is associated with affective characteristics generally insensitive to change [1-3]. Evolutionary theories suggest that affective insensitivity to context or inflexibility to change is an adaptive response that helps preserve energy, a core evolutionary function of depressed mood [3,4]. Unfortunately, contextually insensitive affect appears to have long-term costs for mood, with persistently low reactivity to negative and positive stimuli predicting a poorer depression course with some consistency [5]. Despite these advancements in affective science for understanding depression, these theories have yet to be integrated into clinical practice [6]. Further, clinical practice is still in need of clinical markers that better capture patient-desired outcomes. Current practice guidelines for depression use a symptom management approach to gauge recovery [7]; however, patients place less value on symptom reduction as an outcome and more value on achieving psychological well-being [8]. Based on work consistently showing links between affective flexibility and psychological well-being [2], this paper outlines how clinician-documented affect, which is observed during mental health encounters, may assist in tracking patient-desired depression outcomes over time.

One promising avenue to test this hypothesis is to use data from sequential clinical notes stored in electronic health records (EHRs). EHRs provide temporal information regarding a patient’s affective functioning. As a result, these notes can be used to map a trajectory of outcomes relevant to the depression course. EHRs include significant data from clinical interviews, clinician observations of patients’ affective demeanor, which can capture affective behavior (eg, patient appears unresponsive, incongruent with the emotional content of a verbal report, or agitated), self-reported experiences, and manifestations of physiological affective activation (eg, increased breathing rate and perspiration). Given that affective dysregulation (protracted low mood or anhedonia) is a core aspect of depression, clinicians likely capture relevant affective information in clinical notes and mental health assessment templates, although this is an empirical question that we will revisit in this paper. This information that is already being recorded, through systematic extraction and evaluation, could aid in the development of a measure that incrementally improves how we assess depression, which can ultimately improve clinical practice.

Using data available in the EHR to improve how we capture the depression course and depression outcomes offers mental health care providers a creative solution for bridging clinical science with practice. One way to make use of the EHR and provide a unique opportunity for efficient large-scale investigation is through the development of analytics tools, such as natural language processing (NLP; a method to extract meaningful data from clinical notes) and machine learning (a process to make sense of the data extracted through predictive analyses), both of which can assist with clinical decision-making and make an endeavor, such as the one we are proposing, feasible.

### NLP as a Tool for Understanding Depression

NLP research focuses on developing computational models for understanding natural language [9]. NLP, in its many forms, is used to perform information extraction, which is the extraction of predefined information from texts such as clinical notes [10]. Using tools built around ontologies (controlled vocabularies), like SNOMED-CT (Systematized Nomenclature of Medicine-Clinical Terms) [11], NLP has enabled researchers to automate the capture of information in clinical narratives [10]. This data mining of EHRs can be useful for detecting patterns in patient care, such as choice of treatments, adherence, and changes in functioning and well-being over time [12], which can predict patient treatment habits and their symptom outcomes [13], as well as patient outcomes [14].

The application of analytics tools (eg, NLP) has enabled accurate and efficient determination of longitudinal outcomes. For example, a study by Perlis et al [15] applied NLP using notes from 127,504 patients with a billing diagnosis of major depressive disorder. The NLP classification was developed by a panel of expert clinical psychiatrists who reviewed 5198 patient narrative records to define a classification including 34 terms from the clinical annotations that could distinguish patients who were no longer depressed from those who had treatment-resistant depression. The study found that the NLP models were superior to those relying on billing data alone in classifying a patient’s current mood and longitudinal outcome.

Given that information extraction is based on the extraction of predefined information, the first step in developing a new NLP system is defining the constructs of interest. Specifically, we are interested in what constructs may be relevant to the depression course and outcomes. Currently, the gold standard in clinical practice is monitoring of depression symptom severity. However, this does not adequately capture patient-desired outcomes [8]. Given that depression is conceptualized as an affective disorder based on its 2 core symptoms of protracted negative affect and anhedonia [16], we focused on affective functioning in this study. We expect that affect information would be prevalent in mental health care notes because most major types of therapies for depression tackle some aspect of affective functioning, given that depression is an affective disorder.

### Using Affective Theories of Depression and Empirical Evidence to Guide NLP Development

In this study, we drew from depression and affective theory and research to predefine our main constructs, affect and its characteristics. First, it is important to define the distinctions between emotions and affect. Emotions are immediate and often quick responses to stimuli and environmental changes or events, while affect may be a more diffuse response over the course of minutes and even an hour. Relative to emotions and affect, moods are generally thought to be longer, slower moving, and less tied to specific objects or elicitors [17]. In our analyses, we define affect as encompassing fleeting emotions and states that may be captured during a therapeutic session or clinic visit, while moods are defined as capturing long-lasting affective states that may last days or weeks.

The next key step in developing an NLP tool is understanding how depression may impact affective characteristics. This will help generate a set of adjectives that are expected to be seen in notes when affect is described. For example, anecdotally, people struggling with depression complain that their emotional world is undifferentiated, ﬂat, dull, and empty [18], and behavioral observations from inpatient settings document that depression can diminish motivated activity to the point of immobility, even catatonia in severe cases [19], while others have characterized depression as a syndrome marked by inﬂexibility and stereotypy in cognition, behavior, and physiology [20]. Results from a meta-analysis of 19 laboratory studies on currently depressed individuals revealed a pattern that was termed “emotion context insensitivity” [21,22], in which major depressive disorder was characterized by reduced emotional reactivity to both positively and negatively valenced stimuli, with the reduction larger for positive stimuli (d=−0.53) than for negative stimuli (d=−0.25). Importantly, the meta-analysis revealed similar depression-related differences in emotional reactivity across the following 3 major emotion-response systems: self-reported experience, expressive behavior, and peripheral physiology, reinforcing our current interest in capturing clinician-reported patient affect during clinical sessions. In clinical depression, emotions and affect are often incongruent with mood, in that depressed mood seems to facilitate flat, context insensitive affect, rather than particularly sad emotions [21], a disconnect which seems to strengthen as depression becomes more severe [23].

### Our Study

While the Veterans Affairs (VA) EHR contains information about patient clinical and functional characteristics at the time of a visit, much of it is stored in text notes and is not easily extracted or summarized and readily available to clinicians. Thus, NLP could provide a useful solution to making this information accessible and useable. To facilitate the development of an NLP tool that could be applied to affect information in mental health clinical notes, we conducted a series of 3 pilot studies with the following goals: (1) to identify the major constructs associated with depression as indicated by theory and empirical findings to date; (2) to determine if the targeted constructs can be reliably found in the EHR; and (3) to test the feasibility and reliability of an initial NLP system.

## Methods

### Requesting and Extracting Data

#### Ethics Statement

The Institutional Review Boards at the Department of VA Research and Development (VA R&D) and the University of South Florida approved this study protocol (approval number: IRBPro00029453) and granted waivers of individual consent based on the absence of individually identifying data. The Department of VA provided a waiver of Health Insurance Portability and Accountability Act authorization for research conducted in this study.

#### Data Source and Cohort Selection

The sample for this pilot was extracted from a larger study. We obtained data from the VA Corporate Data Warehouse (CDW), an administrative data source that contains electronic medical records of all Veteran patients who receive care through the VA. The patient cohort included Operation Enduring Freedom/Operation Iraqi Freedom Veteran patients aged 18 to 66 years at the time of their initial elevated Patient Health Questionnaire-9 (PHQ-9) score (≥10) indicative of probable depression, which was recorded anytime during fiscal years 2006 to 2016. During data extraction, we excluded patients who were diagnosed with bipolar disorder, personality disorders, psychotic disorders, and pervasive developmental disorders (to be consistent with the National Committee on Quality Assurance measure of quality of care, the Healthcare Effectiveness Data and Information Set), as well as substance abuse and dependence. The rationale for the exclusion criteria is the divergent treatment practices in the presence of these disorders relative to unipolar depression treatment in the absence of these conditions.

#### Measures

##### Structured Medical Record Data

Patient (N=109) demographic data extracted from the CDW included age, gender, race, and Hispanic ethnicity. Depressive disorder diagnoses and comorbid posttraumatic stress disorder (PTSD), other anxiety disorders, and adjustment disorders were all captured through ICD-9 (International Classification of Diseases, Ninth Revision) codes. PHQ-9 scores obtained during health care visits at the VA were extracted. Finally, we extracted mental health services data associated with a depressive disorder diagnosis, including note and provider type.

##### Unstructured Medical Record Data

Mental health clinical notes (N=147) were randomly selected for our sample of VA (N=109). Two annotators (including ARD) were trained, and they independently annotated each document using Extensible Human Oracle Suite of Tools (eHOST) annotation software developed as part of the VA Consortium for Healthcare Informatics Research project [24]. Notes were grouped into 3 sets so that spot checks could be performed on agreement scores to ensure consistency. An iterative process leading to an agreement of at least 0.70 was used.

### Annotation Guidelines

To support the completion of the annotation process, we developed annotation guidelines and a schema based on depression and affective theories and the literature. The annotation guidelines were developed like those traditionally used for chart review but included more explicit detail about the specific text strings that should be coded (Table 1).

**Table 1:** Affect characteristics as identified in clinical notes.

Construct	Affect	Mood
Theory	Emotion context insensitivity [21]; evolutionary theory [4]	Emotion context insensitivity [21]; evolutionary theory [4]
Definition	A superordinate category for all valenced states [25]. Often used interchangeably with affect is emotion, a subtype of affect, which refers to coordinated responses that occur when an organism encounters meaningful stimuli. Can be indexed by cognitive, experiential, central and peripheral physiological responses, and overt behavior.	Mood is a diffuse construct encompassing emotional experiences (defined by behavioral, psychological, physiological, and cognitive aspects) over the course of days/weeks. It is often not context specific like affect, and it can be impacted by pervasive affect and vice versa.
Characteristics	Descriptive: Blunted, broad, flat, restricted, constricted, less constricted, mood/topic congruent, appropriate to thought/speech content, inappropriate to content, wide ranging, reactive, euthymic, dysthymic, intensity, neither increased nor decreased, irritable, nervous, anxious, sad, angry, tense, labile, within normal limits Behavioral: Crying, laughing, smiling Physiological: Sweating, rapid respiration Timeline of emotional experiences: affect is defined by being observed or expressed during the session.	Descriptive: Depressed/low, dysphoric, anxious, irritable, euphoric, angry Timeline of emotional experiences: mood is defined by a timeline that expands beyond the session.
Examples (from notes)	“patient appeared sad even when talking about his daughter’s accomplishments,” “patient teared up when remembering his lost mother,” “affect is constricted and at times tearful,” “affect is reserved, but reactive,” “affect is tightly controlled, comes across as somewhat blunted,” “affect: tearful at times when appropriate,” “affective expression was serious and melancholic,” “appears sad,” “broader range of affect than yesterday,” “congruent, less flat,” “consistent with mood and topics discussed,” “crying easily,” “difficulty controlling his temper,” “easily tearful,” “easily emotional,” “easily aggravated,” “episodically tearful during the session today,” “expressed frustration and anger multiple times during the assessment,” “feeling emotionally distant,” “his affect seems to be brighter,” “no acute distress noted,” “no emotional response,” “non-labile throughout the session,” “not tearful during the session today,” “patient smiles appropriately,” “poor frustration tolerance,” and “was also able to smile on occasion as appropriate”	“anger/irritable moods,” “anhedonia,” “patient reports anxious mood that varies from daily to weekly,” “anxious, sometimes depressed,” “become anxious in crowds,” “decreased interest,” “devoid of feeling,” “down moods,” “emotional numbing,” “dysphoric/dysthymic,” “emotionally flattening,” “fluctuations in mood,” “feeling unhappy,” “has mood swings (irritable, anxious, depressed),” “he frequently becomes frustrated,” “he has been in a foul mood,” “I am not depressed, I am just angry,” “improved mood,” “inability to express positive emotions,” “increased anxiety over the past few weeks,” “lack of interest in activities,” “lack of pleasure,” “lacks sexual interest,” “less mood instability,” “little interest or pleasure in doing things,” “mood described as *ok*,” “mood has remained somewhat depressed,” “mood is described as *better*,” “mood was neutral,” “not as depressed as he says he feels,” “moody,” “my mood is ok and I am not depressed as much now,” “no mood lability,” and “sustained low/down mood”

#### Affect

Affect is a class that can encompass emotional responses to immediate stimuli and therefore is context dependent. Affect can capture immediate responses as well as responses over the course of minutes to an hour. Affective experience is often multi-component involving behavioral, psychological, physiological, cognitive aspects. This class captures the clinicians’ observations of the patients’ affect (which can be a summation of observations of portrayed emotional behaviors, such as crying and laughing; outward expression of physiological symptoms, such as sweating and rapid respiration; and verbalized cognitions and experiences related to affect, such as expressed distress and worry).

#### Mood

Mood is a diffuse construct encompassing emotional experiences (defined by behavioral, psychological, physiological, and cognitive aspects) over the course of days and weeks. It is often not context specific like affect, and it can be impacted by pervasive affect and vice versa. This class captures patients’ self-report or clinicians’ observations.

### NLP Procedure

As a proof of concept, we conducted a pilot of a simple NLP system using a named entity recognition approach on 147 documents using the final annotations as a reference set. The NLP system, implemented in the Python programming language, first parsed the note for sentences and then checked each sentence for occurrences of the terms or phrases identified as “mood” or “affect” in the text. After the initial NLP extraction, each occurrence was recorded in a data set and compared to the annotated reference set. An error analysis was performed by the clinical psychologist. If an instance found by NLP was not originally in the reference set, but was deemed valid, it was added to the reference set and was counted as a true positive. If it was not a valid instance, it remained a false positive.

## Results

### Structured Medical Record Data

#### Note Characteristics

Out of 147 notes, 64 (43.5%) were written by psychology and 83 (56.5%) by psychiatry mental health providers. The large majority of the notes (119/147, 81.0%) were generated by clinical providers conducting outpatient visits in a VA mental health clinic (ie, specialty clinic), and only 28 (19%) notes were generated in a primary care outpatient setting with an integrated mental health provider. The types of notes are presented in Table 2.

**Table 2:** Types of clinical notes where affect and mood were documented.

Type of note	Value (N=147), n (%)
Mental health consult	16 (10.9)
Mental health note	38 (25.9)
Mental health outpatient note	5 (3.4)
Psychiatry medication management	33 (22.4)
Psychiatry note	27 (18.4)
Psychology note	11 (7.5)
Other types	17 (11.6)

#### Patient Characteristics

The notes included in this pilot were extracted from 109 patients. Patients were on average 40.7 years old (SD 8.2 years; range 27-66 years). Moreover, 100 (91.7%) were male and 57 (52.3%) were married. Clinically, all patients had probable depression (PHQ-9 ≥10). Mean depression severity was 16.1, which is indicative of moderately severe depression (SD 4.1; range 10-25). Diagnostic information suggested that 100 (91.7%) patients had received a depression diagnosis in their charts, 97 (89.0%) had a PTSD diagnosis, and 46 (42.2%) had another anxiety or an adjustment disorder diagnosis.

### Unstructured Medical Record Data

#### Annotation Findings

As noted in Table 3, coding reliability was consistently good across our 3 pilots (range of *F* measure 70%-80%). The language processing data demonstrated that affect characteristics were identified in 123 (83.7%) notes, while mood characteristics were identified in 143 (97.3%) notes. Affect was recorded in 71.9% (46/64) of all notes reported by a clinical psychologist and 92.8% (77/83) of all notes recorded by a psychiatrist. Of those notes reporting any affect during a visit, 37.4% (46/123) were psychology notes and 62.6% (77/123) were psychiatry notes.

**Table 3:** Pilot reliability results.

Variable	Pilot 1 (N=54)		Pilot 2 (N=49)			Pilot 3 (N=44)		
	Percentage present in notes	IAA^a^	Percentage present in notes	IAA	Percentage present in notes		IAA	
Affect	83.3%	79.6%	90.0%	76.1%	90.9%		80.0%	
Mood	94.4%	70.1%	98.0%	76.9%	100%		73.1%	

^a^IAA: interannotator agreement.

#### NLP Accuracy

An overall accuracy of 84.4% was achieved by the NLP system relative to a final reference set of annotations of the 2 concepts. Individually, NLP accuracy in identifying “mood” reached 85.8%, while NLP accuracy in identifying “affect” reached 80.9%.

## Discussion

### Principal Findings

Our preliminary main findings suggest that (1) our theory-driven vocabulary describing affect is indeed captured in patient records with regularity; (2) our vocabulary captured affect in a manner that led to reliable coding by 2 independent coders across 3 pilot samples of clinical notes; and (3) a proof-of-concept NLP system showed good accuracy in capturing affect relative to human coding. Other observations are that affect is frequently present in mental health notes, especially as documented by psychologists and psychiatrists in specialty clinics; clinicians more often documented negative affect, while positive affect was rarely observed in our pilot. Although much of the characteristics were static, in that they were describing emotional expressions specific to the session being documented, on occasion, clinicians did use words suggestive of change, such as comparative adjectives (eg, improved, better, and less).

The pilot findings provide preliminary proof of our concept. Affective characteristics of depressed patients that are theoretically relevant to the depression course and outcomes are frequently reported and reliably identifiable in clinical notes. Our findings showed that clinicians regularly report on patient characteristics that are theoretically and empirically relevant to depression. Although this stored information is not readily accessible to use for outcome research or treatment planning purposes, it appeared to be reported frequently and in a manner that led to reliable coding. Finally, this initial pilot NLP effort showed that we will be able to reliably identify “mood” and “affect” in patients’ medical records.

This work also provided preliminary evidence that affect is described in a variable yet consistent manner, which likely contributed to affect being reliably extracted. Our initial annotation scheme was developed by extracting affect characteristics gleaned from theory and empirical evidence. Both anecdotal accounts [18] and theory of affective functioning in depression [3,4] describe depressed persons reporting their experience of the world as undifferentiated, dull, and generally in a manner that is unresponsive or insensitive to changing contexts. Similarly, many of the patients captured in our pilot data were described as exhibiting “constricted affect, or tightly controlled, coming across as blunted.” Conversely, few patient accounts could be described as labile. Lability of affect appeared more often related to anger, rather than uncontrollable fits of crying spells, for example. Although crying was the most often cited emotional behavior, it was often described as episodic, and the intensity was rarely recorded and mostly implied by therapists’ use of words such as tearful versus crying spell. In future work, it will be important to evaluate whether signs and evidence of reactivity, even when temporary, are related to depression improvement. This work suggests that evidence of emotional reactivity to emotional stimuli is generally related to a more benign course of depression [5]. Finally, positive affect, although theoretically [26] and empirically [2,22] (meta-analyses showing larger effects for positive than negative affect in relation to depression) relevant to the depression state and course [5], was rarely recorded in clinical notes describing patient affect in the session.

The findings that only one-third of the notes documenting affect were written by a psychologist and only two-thirds of all psychologist-written notes documented affect were moderately surprising. This was in part because the gold standard therapy for depression, cognitive behavioral therapy, focuses on developing affective awareness as a major component of the early stages of the therapy. The capacity to distinguish among negative affects is instrumental in the successful deployment of appropriate affect regulation strategies [27]. The fact that psychologists do not always record affect may be important to monitor, especially for studies evaluating consistency in how patients present across clinicians who see patients within the same day. It may also be important to evaluate whether there are qualitative and quantitative differences in the manner that affect is being recorded by psychology and psychiatry notes. Although the high overall accuracy of our NLP tool was reassuring, future work on a larger corpus of notes should look at variability in accuracy based on note characteristics.

Furthermore, although our pilot work was limited in scope, the richness of language observed to be used in clinical notes suggests that tracking *change* will also be feasible on a larger scale. Relevant to capturing affective changes over time that may map onto depression progress and outcomes, we also observed in our annotations language that implied change. Specifically, clinicians used comparative adjectives, such as better and less, to describe observed affective change and record noticeable change from prior sessions. Comparative adjectives will be an important new class of annotations in the next step of this area of research, as they will help with tracking affective and mood changes over time, which are both important to understanding the depression course and possibly depression outcomes.

### Implications of This Work for Understanding and Predicting Depression Outcomes

There is a need for better affective theories that make predictions of the role of affect in the course and outcomes of depression, building on strong evidence showing the implication of affective changes in the course and outcomes of depression [5]. Across various measures of affect, both reduced positive affect and reduced state negative affect predicted a poor depression course. Based on prior work, greater affective responsiveness to sad [21] and amusing [23] laboratory stimuli predicted a more benign course of major depression. Conversely, lack of affective response predicted long-term heightened depression severity. For example, greater endorsement of positive, but not negative, words predicted depressive symptoms 9 months later [28], and people with depression exhibiting the lowest behavioral reactivity to an amusing film showed worse depression severity 1 year later [23]. Affective reactivity to daily life events was predictive of symptomatic change at 1 month after treatment [29]. This body of work suggests that signs of positive and negative affect dysregulation are likely to be germane to the prediction of long-term outcomes in depression.

Finally, affective characteristics also appear to fluctuate with the depressed state and history, such that remitted depressed individuals look more like healthy controls than currently depressed individuals on various measures of affective reactivity (measured through physiological and behavioral measures) [30,31]. Prospective links between affect and depression outcomes make good sense within a functionalist perspective on affect [32], given that affect represents dynamic adjustments to environmental challenges and opportunities across time. Nevertheless, we do not yet know how observable affective characteristics within the context of naturalistic mental health visits are important in predicting the depression course in the context of mental health care. While other studies have demonstrated success using NLP to detect treatment-resistant depression [15], we propose that future work may build on our findings by specifically relating affect-related measures to depression outcomes in adults.

### Future Directions

Despite research indicating the potential for improvements in depression outcomes, there is a dearth of research on outcome enhancement through the use of analytics tools. Using NLP to develop methods to reliably extract the routine documentation of affect by mental health care providers in clinical notes and structure it for use in health services research is a foundational step. We would like to offer the following suggestions for future research that could help this field move forward:

1. The development of NLP analysis that may predict depression outcomes is the ideal extension of this work. Hence, the evaluation of affect over time is a crucial next step. Specifically, the next meaningful question is as follows: Are we able to detect meaningful change in affect across sequential notes? Practically, is affect reported and recorded in a way that will translate to an observable and clinically meaningful change? Extraction of affect over time within the patient is key for this step. Based on the literature reviewed in this proof of concept and our pilot work, we believe this will be a fruitful endeavor.
2. Another necessary step entails validation of our “measure.” Does our measure converge with measures we assume it intersects with, such as measures of depression symptoms, well-being, and functioning? Such work not only would provide a preliminary validation for this clinical tool, but also may highlight potential discriminant validity.
3. To further validate this tool, collection of patient behaviors during clinical sessions and evaluation of the alignment or misalignment with provider coding of affect would initiate an evaluation of the pieces of information that providers account for when coding a patient’s affective state during a session.
4. Ultimately, our work sets the preliminary steps to developing an NLP tool. Automating the extraction and interpretation of affective information from mental health session notes would provide a monitoring tool that could benefit patients and clinicians alike by highlighting clinically meaningful changes. This information could inform clinical decision-making.

## Abbreviations

CDW: Corporate Data Warehouse
eHOST: Extensible Human Oracle Suite of Tools
EHR: electronic health record
NLP: natural language processing
PHQ-9: Patient Health Questionnaire-9
PTSD: posttraumatic stress disorder
VA: Veterans Affairs

## Data Availability

Data are not available to share due to Department of Veterans Affairs and ethical retractions; however, annotation instructions and vocabulary are available upon request.
